# Sexual dimorphism alters seasonal chelae muscle mechanisms in spiny-cheek crayfish (*Faxonius limosus*)

**DOI:** 10.3389/fphys.2025.1567862

**Published:** 2025-04-02

**Authors:** Przemysław Śmietana, Natalia Śmietana, Piotr Eljasik, Sławomir Lisiecki, Małgorzata Sobczak, Remigiusz Panicz

**Affiliations:** ^1^ Department of Environmental Ecology, Institute of Marine and Environmental Sciences, University of Szczecin, Szczecin, Poland; ^2^ Faculty of Food Sciences and Fisheries, West Pomeranian University of Technology Szczecin, Szczecin, Poland

**Keywords:** freshwater ecosystem, gene expression, mating behaviour, molecular mechanism, principal component analysis

## Abstract

Sex-specific behaviours of freshwater crayfish are key elements in sustaining species persistence and successful conquering of new habitats in freshwater ecosystems. However, to date, information on molecular mechanisms that underpin the anatomy and physiology of crayfish sexes in successful mating behaviour was scarcely presented. In this study, *Faxonius limosus* females and males were sampled in spring and autumn to assess the impact of sexes and seasons on body parameters and activity of *arginine kinase* (*ak*), *ferritin* (*fr*), *crustacean calcium-binding protein 23* (*ccbp-23*), *troponin c* (*tnnc*), and *skeletal muscle actin 8* (*actinsk8*) genes related to the functioning of muscles in chelae. Comparison of body parameters showed significant differences in the weight and size of individuals in two seasons, underlining that large chelae are essential for males in mating behaviours and male-male competitive interactions. The gene expression analysis showed that activities of the five genes in the chelae muscle of *F. limosus* were influenced by the season- and sex-specific drivers. Multivariate analyses specifically identified the key genes (e.g., *tnnc* in males from spring) that were directly involved in metabolisms of chelae muscles of males and females collected in spring and autumn. The study, for the first time, described the direct impact of two key seasons and sexes on the anatomical features and molecular mechanisms that shaped the behaviour of *F. limosus*.

## 1 Introduction

Freshwater crayfish exhibit pronounced sexual dimorphism in both their anatomy and behaviour. The differences are related to the distinct roles each sex plays in mating and reproduction (Śmietana, 2013). During the mating season, mature males actively explore inhabited areas and seek for sexually-mature females that may be identified directly (antennae, claws) and indirectly (pheromones) (Zhou et al., 2023). The mating process itself requires significant effort from the males, as it involves a combination of display behaviours (e.g., aggressive movements such as claw waving or chasing), flipping the female on the dorsal side, and holding the female down with claws to secure position for an extended period. Additionally, males fiercely compete with other wooers by physically grappling to mate with possibly the highest number of females that co-inhabit the benthic zone (Kubec et al., 2019). While females remain relatively passive, and those ready to reproduce simply do not flee from males (Kozák et al., 2015). After the breeding season, males switch back to the typical non-mating behaviour, while females enter the most exhausting and energy-demanding period that includes egg development, laying, and incubation, along with the initial “care” of the offspring until disconnected after the first post-embryonic moulting. During the period of egg carrying, females direct their main activity to maintain the eggs clean and oxygenated and significantly minimise general activity to obtain food. After this stage, females become more active as they need to maximise food intake and reestablish depleted energy resources. The differences in reproduction behaviour substantially affect energy management in both sexes which leads to allometric growth and unsynchronised moulting events. Moreover, differences in growth rates represent another aspect of sexual dimorphism between crayfish sexes, additionally exemplified by the larger size of the claws in males and broader abdomen in females (Kozák et al., 2015; Reynolds, 2002).

The mating-oriented life cycle, as well as apparent sexual characteristics in anatomy and behaviour in males and females of spiny-cheek crayfish (*Faxonius limosus*), are presumably interconnected with molecular mechanisms that underpin different metabolism rates of chelae muscles across seasons. There are no studies that would demonstrate the effect of seasons on the expression of genes related to the development and contraction of muscles in the chelae of spiny-cheek crayfish and how the seasonal behaviours may be mirrored in those activities. Moreover, *F. limosus* is a well-known non-native and invasive crayfish in the European freshwater system, but to date, the number of molecular studies covering this species is still scarce. Currently, much-needed novel genetic methods for the control of invasive species are under development (Teem et al., 2020). However, prior knowledge of molecular mechanisms is required to precisely target innate mechanisms and develop strategies that will ensure effective management or eradication strategies.

Therefore, this study aimed to determine how seasonal changes influence the expression and role of five genes directing the metabolism of chelae muscles (*skeletal muscle actin 8*, *arginine kinase*, *ferritin*, *crustacean calcium-binding protein 23*, and *troponin c*) in females and males of invasive spiny-cheek crayfish considering the sex of individuals and two seasons. The hypothesis of the study states that males and females will exhibit distinct metabolic patterns across different seasons that are key in the life cycle of this species. The study will provide unique information on innate molecular mechanisms in males and females of *F. limosus* that may pave the way for new strategies to efficiently manage populations of invasive crayfish.

## 2 Materials and methods

### 2.1 Sample collection and morphometric measurements

Adult males (n = 5 per season) and females (n = 5 per season) of spiny-cheek crayfish were caught during two sampling seasons, i.e., spring (May) and autumn (September), from Sominko Lake (Northwest Poland, 54°04′46″N, 17°52′48″E) by scuba diving and manual picking. Each time, crayfish were collected during the daytime from the same part of the lake, within a 100 m stretch from the shoreline at depths ranging from 0.20 to 4.5 m to minimise environmental impact on gene expression. Consequently, the presence of spiny-cheek crayfish was initially assessed in all types of shelters, including burrows, branches, and objects lying on the bottom, such as wooden planks. Females caught in the spring were carrying eggs and were in the second half of their incubation period (approximately 1 week before the offspring hatched). In the autumn, both males and females were captured at the beginning of the mating season. The captured individuals were placed in a container with water at approximately 4°C and transported to the laboratory. In the laboratory, crayfish were anesthetised and sacrificed using low temperature, following general advice on animal welfare (Faculty Ethical Committee number 517-08-026-7724/17). Next, individuals were weighed (to the nearest 0.01 g) using laboratory balance WLC/C/2 (Radwag, Poland), total body length (TL) and claw length (CL) were measured (to the nearest 0.01 cm) using an electronic calliper, and CL/TL ratio was calculated.

### 2.2 Gene expression and statistical analysis

Subsequently, males and females from each season were sampled for gene expression analysis, following the detailed procedure described in Śmietana et al. (2020). Briefly, the muscles from both chelae were individually removed and homogenised in 750 µL of TRI Reagent L203 (Zymo Research, United States). Next, total RNA was extracted using Direct-zol RNA Miniprep Kit (Zymo Research, United States) and reverse transcription was performed using 500 ng of total RNA with Transcriptor First Strand cDNA Synthesis Kit (Roche, Switzerland). The qPCR was performed in duplicates for five muscle metabolism-related genes i.e., *skeletal muscle actin 8* (*actinsk8*), *arginine kinase* (*ak*), *ferritin* (*fr*), *crustacean calcium-binding protein 23* (*ccbp-23*), and *troponin c* (*tnnc*). Relative gene expression was assessed against two reference genes, i.e., *eukaryotic translation initiator 5a* and *β-actin*, using the 2^−ΔΔCt^ method (Livak and Schmittgen, 2001). Statistical analyses were performed using Statistica 13 software (TIBCO Software inc., USA). Normality distribution was assessed using the Shapiro-Wilk test, and the significance of the observed differences was assessed using non-parametric tests i.e., Mann Whitney U-Test (pairwise) or one-way non-parametric ANOVA (Kruskal–Wallis test) with a two-sided Bonferroni adjustment *post hoc* test. Principal Component Analyses (PCA) were conducted to show the seasonal variations depending on sex. Data were visualised using the ggplot2 in R (Wickham, 2016).

## 3 Results

### 3.1 Morphometric measurements

**FIGURE 1:**
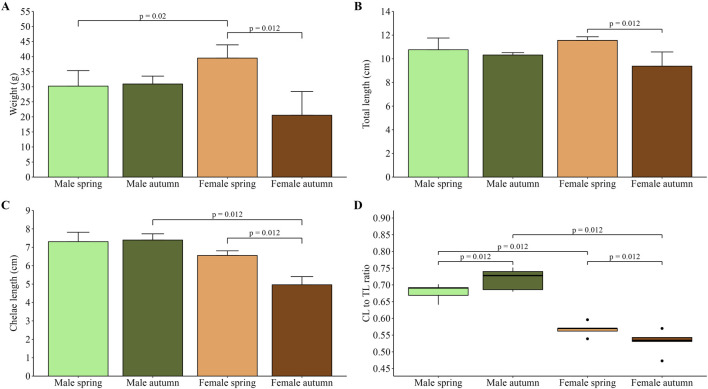
Average weight of male and female spiny-cheek crayfish collected in spring and autumn **(A)**; total body length **(B)** and chelae length **(C)** of male and female crayfish in two seasons; CL/TL ratio of male and female *F. limosus* caught in two seasons **(D)**. The significance of differences was assessed using Kruskall-Wallis test with a two-sided Bonferroni adjustment *post hoc* test.

Body weight comparison revealed that during spring, females were significantly (p = 0.02) heavier than males, while in autumn, no difference (p = 0.09) was observed (Figure 1A). In the case of males, no difference was found between the two seasons (p = 0.90), whereas females caught in spring were significantly heavier compared to those caught in autumn (p = 0.012). Consequently, the greatest TL was observed in females caught in the spring; however, the difference was statistically insignificant (p = 0.40) when compared to males from the same season (Figure 1B). Moreover, no difference was found between males across the seasons, while a significant difference was shown for females (p = 0.012). Chelae length measurements showed that females in autumn had the lowest CL (Figure 1C). The difference was significant when compared to females caught in spring (p = 0.012) and males collected in autumn (p = 0.012), which had the greatest CL. No difference (p = 0.06) was found in CL between both sexes in spring. Additionally, differences between sexes in CL and TL were further highlighted in CL/TL ratio. Analysis showed that males in both seasons (spring and autumn) had a significantly greater CL/TL ratio (p = 0.012 for all comparisons) compared to females in the respective season (Figure 1D). Moreover, significant differences were found between males across seasons (p = 0.012) and between females caught in two seasons (p = 0.012).

### 3.2 Gene expression analysis

**FIGURE 2:**
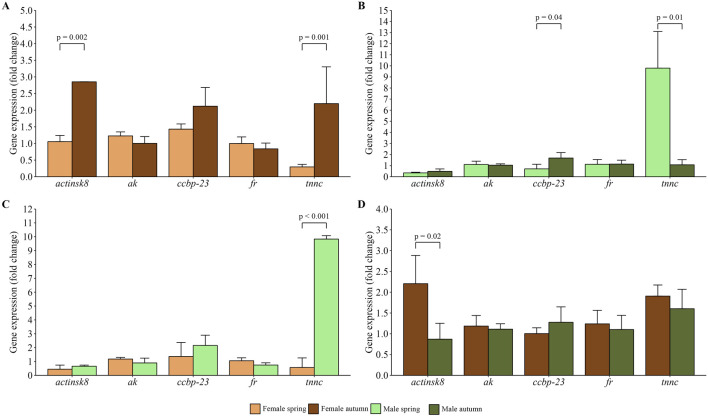
Relative gene expression profiles in chelae muscles of females **(A)** and males **(B)** spiny-cheek crayfish (*Faxonius limosus*) across spring and autumn seasons, and differences in gene expression profile between females and males in spring **(C)** and autumn **(D)**. Gene expression was assessed against two reference genes, i.e., *eukaryotic translation initiator 5a*, and *β-actin*. The significance of differences was assessed using Mann-Whitney U test. Abbreviations: *arginine kinase* (*ak*), *ferritin* (*fr*), *crustacean calcium-binding protein 23* (*ccbp-23*), *troponin c* (*tnnc*), *skeletal muscle actin 8* (*actinsk8*).

Gene expression analysis showed significantly higher relative expression of *actinsk8* (p = 0.002) and *tnnc* (p = 0.001) genes in chelae of females caught in autumn compared to those caught in spring, while no differences were noted in *ak*, *ccbp23*, and *fr* expression (Figure 2A). In the case of males, differences were noted in *ccbp-23* and *tnnc* mRNA transcripts number in chelae muscle between spring and autumn samplings (Figure 2B). More specifically, *tnnc* expression was higher in spring (p = 0.01), *ccbp-23* expression was higher in autumn (p = 0.04), whereas the activity of remaining genes was at a similar level. Comparison of males and females caught in spring showed significantly higher (p = 0.001) expression of *tnnc* in male chelae muscle (Figure 2C). In autumn, *ak*, *ccbp-23*, *and fr* expression in chelae muscle revealed a similar pattern to spring when comparing males with females caught in the season (Figure 2D). However, *actinsk8* and *tnnc* expression showed a reverse pattern compared to spring, with the former being significant (p = 0.02) in comparison of males and females collected in autumn.

**FIGURE 3:**
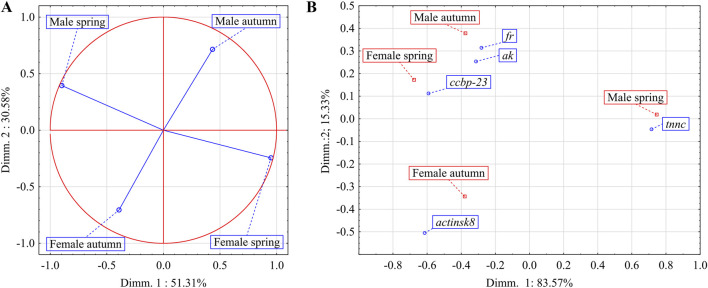
The impact of sex and season on the overall expression profiles of *ak*, *actinsk8*, *ccbp-23*, *fr,* and *tnnc genes* in chelae muscles of spiny-cheek crayfish (*Faxonius limosus*) **(A)**; main determinants (genes expressed in chelae muscles) of differences between males and females of *F. limosus* in spring and autumn **(B)**. Abbreviations: *arginine kinase* (*ak*), *ferritin* (*fr*), *crustacean calcium-binding protein 23* (*ccbp-23*), *troponin c* (*tnnc*), *skeletal muscle actin 8* (*actinsk8*).

The correlation circle for PCA showed the strongest (negative) correlation for males and females from spring as well as for males and females from autumn (Figure 3A). Furthermore, the correspondence analysis showed that expression of *ak* and *fr* genes had the most profound effect on the uniqueness of males group collected in autumn from other spiny-cheek crayfish groups. Whereas the males collected in spring were the most distinctive due to the activity of the *tnnc* gene (Figure 3B; Table 1). In the case of females, the difference between the spring and autumn group of females was significantly smaller than for male groups (15.33% vs. 83.57%). Females collected in autumn and females in spring had a distinctively higher activity of *actinisk8* and *ccbp-23* than the other groups, respectively. Further, both groups of females shared comparable expression profiles to the males captured in autumn.

**TABLE 1:** The PCA coordinates indicating the contribution and direction imparted by the expression levels of individual genes in chelae muscles of spiny-cheek crayfish (*Faxonius limosus*) in Dimensions 1 and 2.

Gene	Coordinates of dimension 1	Coordinates of dimension 2
*Arginine kinase* (*ak*)	−0.313446	0.253686
*Calcium-binding protein 23* (*ccbp-23*)	−0.591487	0.111721
*Ferritin* (*fr*)	−0.281355	0.313905
*Skeletal muscle actin 8* (*actinsk8*)	−0.614241	−0.505240
*Troponin c* (*tnnc*)	0.715138	−0.046474

## 4 Discussion

### 4.1 Sexual dimorphism in species biology across seasons

In crayfish biology, seasonality is the main driver that constantly and consequently affects all elements of their lifecycle and is mainly directed to mating, reproduction and initial offspring care. Those activities are evolutionary-rooted, and the successful persistence of crayfish populations is largely affected by sexual dimorphism. In this study, a greater body weight of females compared to males in spring may be attributed to two phenomena. First, in the spring, females are carrying and incubating eggs attached to their abdomens, which naturally increase the total body weight measured. Second, the largest females are the easiest to catch in the spring since they tend to occupy the shoreline, where warm and well-oxygenated surface water accelerates egg incubation. Smaller females, due to strong competition, are forced to hide in deeper layers of lakes, and thus less accessible for sampling by scuba diving (Śmietana, 2013). This sampling method might have possibly caused a bias. However, if baited trap sampling was applied, the largest individuals were collected at the beginning and during the subsequent attempts the size of the entrapped crayfish decreased (Holdich and Black, 2007). Moreover, traps are highly selective and ineffective compared to scuba sampling which is currently the most representative and effective method applied in crayfish sapling. In the autumn, there is no tendency for large females to inhabit the littoral zone, therefore, caught females were significantly lighter compared to those from the spring. The lack of differences between males and females in autumn could be influenced by the high variability (min = 12.46 g, max = 31.31 g) within the female samples collected in autumn, as well as the small sample size (n = 5). Nevertheless, these results may suggest that females do not show as strong habitat preference in the autumn as in the spring (Buřič et al., 2009). Found in our study, differences in body weight and TL, and especially the greatest body length of females in spring, are in line with Reynolds (2002), who reported the allometric growth in crayfish, where the development of the female abdomen is prioritised over other parts when approaching sexual maturity.

The chelae size plays an important role in active food acquisition and defence against predators of male spiny-cheek crayfish (Buřič et al., 2010). Moreover, chelae size and strength are essential in the “sperm competition” tactic, which is a powerful selective force in numerous species (Keller and Hazlett, 1996; Yue et al., 2010). Large chelae bear a higher number of receptors that decide on the detection efficiency of females’ pheromones. Belanger and Moore (2006) showed that rusty crayfish (*Faxonius rusticus*) males with more developed chelae have a larger proportion of plumose and simple setae, capable of chemo- and mechano-sensing. In our study, no difference was found in claw length between both sexes in spring; however, there is a substantial likelihood of encountering a Type II error due to a small sample size. Moreover, the results of CL/TL ratio comparisons confirm that large chelae are essential for males in the breeding period (autumn) and in male-male competitive interactions, which eventually affect breeding success (Goessmann et al., 2000; Yue et al., 2010). Furthermore, larger chelae size observed in females from non-breeding (spring) compared to breeding (autumn) period may be explained by a more critical competitive ability to defend shelters (burrows) against other females, while smaller chelae in the breeding season could be linked with plausible energy channelling towards egg development (Heuring and Hughes, 2019).

### 4.2 Gene activities reflect sex- and season-specific mechanisms

Morphological and behavioural differences between crayfish sexes result from the cellular and molecular processes, which in turn directly depend on seasonal fluctuations of environmental stimuli. In crayfish, highly active chelipeds play numerous and specific roles across different seasons and to a large extent, support population persistence. However, until now, no studies have been performed to identify changes in gene activities in muscles of the most active part of chelipeds, i.e., chelae, considering non-breading and breading periods of the year and inherent differences between *F. limosus* sexes. Although only five males and five females per season were analysed our study showed significant differences in gene expression profiles, as shown for another crayfish species *Cherax quadricarinatus* (Lu et al., 2021). The higher expression of *actinsk8* in females during the autumn season implicates season-related activity. The active defensive behaviour of females towards males upregulates *actinsk8* gene activity, that codes the actin isoform SK8 - a “fast-type” actin involved in the signalling and responsiveness of contraction mechanism (Gordon et al., 2000; Kim et al., 2009). Aquiloni and Gherardi (2008) showed that red swamp crayfish (*Procambarus clarkii*) females use chemical stimuli to attract the largest (most suitable) mate, which are sensed through male’s chelae. Furthermore, actins are known to be sensitive to non-genomic signalling (e.g., hormonal) thus alleviated *actinsk8* gene expression in female spiny-cheek crayfish might be caused by males’ proximity (Stournaras et al., 2014; Yazicioglu et al., 2016). Alternatively, but not exclusively, our findings indicate that increased *actinsk8* expression in autumn may be linked to enhanced foraging behaviour before winter, potentially affecting energy reserves for the reproductive period (Kozák et al., 2015). Overall, the results in chelae are also in line with previously reported variability of *actinsk8* expression in the abdomen of *F. limosus* sampled during the spring and autumn seasons (Śmietana et al., 2020). Troponin C is a protein that, together with troponin I and T, forms a heterotrimeric troponin complex that induces muscle cell contraction by regulating the interaction between actin and myosin through Ca^2+^ signalling (Haiech et al., 2019). The elevated expression of *tnnc* in male *F. limosus* during spring may indicate muscle preparation for mating-related competitive interactions. For instance, higher *tnnc* expression suggests either regeneration of muscles after the intense breeding season or muscle atrophy (growth) before moulting (Buřič et al., 2013), since excitation of Ca^2+^ signalling pathway was observed in pre-moulting (ecdysis) stage of crustaceans (Mykles, 2021). In the case of females, overexpression of *tnnc* in autumn was most probably linked with food acquisition (Kozák et al., 2015), similarly to *actinsk8* activity. The CCBP-23 also plays an important role in Ca^2+^ signalling, and similarly to other calcium-binding proteins, regulates muscle contraction (Sauter et al., 1995). The observed in our study difference in the expression of *ccbp-23* may not have a direct ecological explanation since the Ca^2+^ signalling pathway is inherently reactive and responds to various external stimuli (Mykles, 2021). Our results showed that the remaining two genes expression, i.e., *fr* and *ak* were not affected by seasonal changes in both males and females of spiny-cheek crayfish. This result indicates a more general role (e.g., regulation of cellular homeostasis) of *ferritin* in chelae and the physiological role of *arginine kinase* in muscle functioning, osmotic balance, and energy metabolism (Durand et al., 2004; Shofer et al., 1997).

The PCA confirmed the observations discussed above that the seasonal characteristics play a pivotal role in the control of the crayfish life cycle and this impact may be characterised by assessing the level of gene activities in the muscle of claws in males and females of spiny-cheek crayfish. The undeniable impact of the environmental variability on gene expression levels is exemplified by the clear and opposite hierarchical arrangement of groups on the axes. Specifically in spring, elevated expression was observed for different genes than in autumn, representing a diametrical change in the hierarchy of genes in the seasons. Additionally, the PCA plot showed that sex of the crayfish determines expression profiles of the studied genes and that the nature of this influence differs from that observed for the impact of the season, i.e., the hierarchical arrangement remains the same concerning the order of expression but is reversed. This means that when the expression of a particular gene reaches its maximum in males, it will exhibit a minimal level in females. Furthermore, the results of the correspondence analysis showed that the activity of genes involved in the regulation of claw muscle is directly related to the sex-specific reproduction strategies of *F. limosus*. The profound distinctiveness of the males collected during the spring season against other groups provides sound evidence for that unique observation.

Freshwater crayfish are recognised to play key roles in ecosystem functioning due to its involvement in the decomposition of organic matter, nutrient cycling and ecological associations such as communities and biomes. Here, we presented that non-breading (spring) and breading (autumn) seasons triggered sex-specific behaviours of *F. limosus,* which directly affected the size of claws and abdomen in females and males. Our study also showed that the activities of *ak*, *actinsk8*, *ccbp-23*, *fr*, and *tnnc* genes in chelae muscles were influenced by the season-specific conditions and sex of spiny-cheek crayfish. Further, the multivariate analyses, PCA and correspondence analysis identified individual genes directly involved in the sex- and season-dependent metabolism of claw muscles. While this study demonstrates significant seasonal and sex-specific molecular changes in chelae muscle of *F. limosus*, further investigations are necessary to establish its applicability as a model organism. Further research should include a higher number of biological samples, target a higher number of genes (e.g., through implementing RNA-seq) and identify differentially expressed and coregulated genes considering different environmental variables, such as water temperature, predation pressure, and pollution, to confirm seasonal patterns observed in our work. Additional findings will find links between gene expression profiles and behavioural adaptation of crayfish and will eventually assist procedures for crayfish population management focused on invasive species control or species-specific conservation strategies.

## Data Availability

The original contributions presented in the study are publicly available. This data can be found here: https://doi.org/10.5281/zenodo.15037862.

## References

[B1] Aquiloni L. Gherardi F. (2008). Assessing mate size in the red swamp crayfish *Procambarus clarkii*: effects of visual versus chemical stimuli. Freshw. Biol. 53 (3), 461–469. 10.1111/j.1365-2427.2007.01911.x

[B2] Belanger R. M. Moore P. A. (2006). The use of the major chelae by reproductive male crayfish (*Orconectes rusticus*) for discrimination of female odours. Behaviour 143, 713–731. 10.1163/156853906777791342

[B3] Buřič M. Kouba A. Kozák P. (2010). Intra-sex dimorphism in crayfish females. Zoology 113 (5), 301–307. 10.1016/j.zool.2010.06.001 20932733

[B4] Buřič M. Kouba A. Kozák P. (2013). Reproductive plasticity in freshwater invader: from long-term sperm storage to parthenogenesis. PLOS ONE 8 (10), e77597. 10.1371/journal.pone.0077597 24204886 PMC3804581

[B5] Buřič M. Kozák P. Kouba A. (2009). Movement patterns and ranging behavior of the invasive spiny-cheek crayfish in a small reservoir. Arch. Hydrobiol. 174 (4), 329–337. 10.1127/1863-9135/2009/0174-0329

[B6] Durand J. P. Goudard F. Pieri J. Escoubas J. M. Schreiber N. Cadoret J. P. (2004). Crassostrea gigas ferritin: cDNA sequence analysis for two heavy chain type subunits and protein purification. Gene 338 (2), 187–195. 10.1016/j.gene.2004.04.027 15315822

[B7] Goessmann C. Hemelrijk C. Huber R. (2000). The formation and maintenance of crayfish hierarchies: behavioral and self-structuring properties. Behav. Ecol. Sociobiol. 48, 418–428. 10.1007/s002650000222

[B8] Gordon A. M. Homsher E. Regnier M. (2000). Regulation of contraction in striated muscle. Physiol. Rev. 80 (2), 853–924. 10.1152/physrev.2000.80.2.853 10747208

[B9] Haiech J. Moreau M. Leclerc C. Kilhoffer M. C. (2019). Facts and conjectures on calmodulin and its cousin proteins, parvalbumin and troponin C. Biochim. Biophys. Acta Mol. Cell Res. 1866 (7), 1046–1053. 10.1016/j.bbamcr.2019.01.014 30716407

[B10] Heuring W. L. Hughes M. (2019). It takes two: seasonal variation in sexually dimorphic weaponry results from divergent changes in males and females. Ecol. Evol. 9 (9), 5433–5439. 10.1002/ece3.5136 31110691 PMC6509379

[B11] Holdich D. Black J. (2007). The spiny-cheek crayfish, *Orconectes limosus* (Rafinesque, 1817)[Crustacea: Decapoda: cambaridae], digs into the UK. Aquat. Invasions 2 (1), 1–15. 10.3391/ai.2007.2.1.1

[B12] Keller T. A. Hazlett B. A. (1996). Mechanical use of crayfish chelae. Mar. Freshw. Behav. Physiol. 28 (3), 149–162. 10.1080/10236249609378986

[B13] Kim B. K. Kim K. S. Oh C. W. Mykles D. L. Lee S. G. Kim H. J. (2009). Twelve actin-encoding cDNAs from the American lobster, *Homarus americanus*: cloning and tissue expression of eight skeletal muscle, one heart, and three cytoplasmic isoforms. Comp. Biochem. Physiol. B 153 (2), 178–184. 10.1016/j.cbpb.2009.02.013 19258044

[B14] Kozák P. Duris Z. Petrusek A. Buřič M. Horká I. Kouba A. (2015). Crayfish biology and culture. Ceské Budějovice. Vodňany, Czech Republic: University of South Bohemia, 456.

[B15] Kubec J. Kouba A. Buřič M. (2019). Communication, behaviour, and decision-making in crayfish: a review. Zool. Anz. 278, 28–37. 10.1016/j.jcz.2018.10.009

[B16] Livak K. J. Schmittgen T. D. (2001). Analysis of relative gene expression data using real-time quantitative PCR and the 2(-Delta Delta C(T)) Method. Methods 25 (4), 402–408. 10.1006/meth.2001.1262 11846609

[B17] Lu Y. P. Zheng P. H. Zhang X. X. Wang L. Li J. T. Zhang Z. L. (2021). Effects of dietary trehalose on growth, trehalose content, non-specific immunity, gene expression and desiccation resistance of juvenile red claw crayfish (*Cherax quadricarinatus*). Fish and Shellfish Immunol. 119, 524–532. 10.1016/j.fsi.2021.10.043 34737131

[B18] Mykles D. L. (2021). Signaling pathways that regulate the crustacean molting gland. Front. Endocrinol. 12, 674711. 10.3389/fendo.2021.674711 PMC8256442 34234741

[B19] Reynolds J. D. (2002). “Growth and reproduction,” in Biology of freshwater crayfish. Editor Holdich D. M. (Oxford: Blackwell Science), 152–191.

[B20] Sauter A. Staudenmann W. Hughes G. J. Heizmann C. W. (1995). A novel EF-hand Ca2+-binding protein from abdominal muscle of crustaceans with similarity to calcyphosine from dog thyroidea. Eur. J. Biochem. 227 (1–2), 97–101. 10.1111/j.1432-1033.1995.tb20363.x 7851448

[B21] Shofer S. L. Willis J. A. Tjeerdema R. S. (1997). Effects of hypoxia and toxicant exposure on arginine kinase function as measured by 31P-NMR magnetization transfer in living abalone. Comp. Biochem. Physiol. C 117 (3), 283–289. 10.1016/S0742-8413(97)00007-8

[B22] Śmietana N. Panicz R. Sobczak M. Eljasik P. Śmietana P. (2020). Validation of real-time PCR reference genes of muscle metabolism in harvested spiny-cheek crayfish (*Faxonius limosus*) exposed to seasonal variation. Animals 10 (7), 1140–1213. 10.3390/ani10071140 32640616 PMC7401605

[B23] Śmietana P. (2013). Distributional conditionings and interspecific competition of the noble crayfish (*Astacus astacus* L.) and the spiny-cheek crayfish (*Orconectes limosus* Raf.) in the waters of Pomerania. Szczec. Wydaw. Nauk. Uniw. Szczecińskiego. Rozpr. i Stud. – Szczecin, Poland: Uniw. Szczeciński 860, 214.

[B24] Stournaras C. Gravanis A. Margioris A. N. Lang F. (2014). The actin cytoskeleton in rapid steroid hormone actions. Cytoskeleton 71 (5), 285–293. 10.1002/cm.21172 24616288

[B25] Teem J. L. Alphey L. Descamps S. Edgington M. P. Edwards O. Gemmell N. (2020). Genetic biocontrol for invasive species. Front. Bioeng. Biotechnol. 8, 452. 10.3389/fbioe.2020.00452 32523938 PMC7261935

[B26] Wickham H. (2016). ggplot2: Elegant Graphics for Data Analysis. Springer Cham. 260. 10.1007/978-3-319-24277-4

[B27] Yazicioglu B. Reynolds J. Kozák P. (2016). Different aspects of reproduction strategies in crayfish: a review. Knowl. Manag. Aquat. Ecosyst. 417, 33. 10.1051/kmae/2016020

[B28] Yue G. H. Le Li J. Wang C. M. Xia H. Wang G. L. Feng J. B. (2010). High prevalence of multiple paternity in the invasive crayfish species, *Procambarus clarkii* . Int. J. Biol. Sci. 6 (1), 107–115. 10.7150/ijbs.6.107 20186292 PMC2828620

[B29] Zhou Z. Wu H. Wu Z. Mo L. Li D. Zeng W. (2023). Identification of sex pheromone of red swamp crayfish *Procambarus clarkii* and exploration of the chemosensory mechanism of their antennae. Pestic. Biochem. Physiol. 195, 105580. 10.1016/j.pestbp.2023.105580 37666605

